# *Arabidopsis* Non-Coding RNA Regulation in Abiotic Stress Responses

**DOI:** 10.3390/ijms141122642

**Published:** 2013-11-18

**Authors:** Akihiro Matsui, Anh Hai Nguyen, Kentaro Nakaminami, Motoaki Seki

**Affiliations:** 1 Plant Genomic Network Research Team, RIKEN Center for Sustainable Resource Science, 1-7-22 Suehiro-cho, Tsurumi-ku, Yokohama, Kanagawa 230-0045, Japan; E-Mails: akihirom@psc.riken.jp (A.M.); nguyenhaianh@psc.riken.jp (A.H.N.); nm_ken@psc.riken.jp (K.N.); 2 Kihara Institute for Biological Research, Yokohama City University, 641-12 Maioka-cho, Totsuka-ku, Yokohama, Kanagawa 244-0813, Japan; 3 CREST, JST, 4-1-8 Honcho, Kawaguchi, Saitama 332-0012, Japan

**Keywords:** non-coding RNA, small RNA, antisense RNA, abiotic stress response

## Abstract

Plant growth and productivity are largely affected by environmental stresses. Therefore, plants have evolved unique adaptation mechanisms to abiotic stresses through fine-tuned adjustment of gene expression and metabolism. Recent advanced technologies, such as genome-wide transcriptome analysis, have revealed that a vast amount of non-coding RNAs (ncRNAs) apart from the well-known housekeeping ncRNAs such as rRNAs, tRNAs, small nuclear RNAs (snRNAs) and small nucleolar RNAs (snoRNAs) are expressed under abiotic stress conditions. These various types of ncRNAs are involved in chromatin regulation, modulation of RNA stability and translational repression during abiotic stress response. In this review, we summarize recent progress that has been made on ncRNA research in plant abiotic stress response.

## 1. Introduction

Environmental stresses, such as drought, heat, salinity and low temperature, are major limiting factors for plant growth and productivity. Under natural conditions, plants are exposed to a variety of environmental stresses. In order to adapt and survive under the stresses, plants have evolved various molecular mechanisms for a fine-tuned control of adaptive responses [1]. Post-transcriptional regulatory mechanisms, as well as epigenetic and post-translational modifications, like ubiquitination and sumoylation, have been implicated to play an important role in the regulation of gene expression during stress conditions.

Recent genome-wide transcriptome analysis, such as tiling arrays and next generation sequencing, has revealed a large number of stress-responsive ncRNAs. Emerging evidence has revealed that ncRNAs are major products of the plant transcriptome with significant regulatory importance [2,3]. ncRNAs are transcribed from intergenic regions, antisense strands of protein-coding genes and also pseudogenes. According to their size, ncRNAs are classified as small ncRNAs (sRNAs) (<40 nt) and long ncRNAs (lncRNAs) (>200 nt). These ncRNAs are involved in the transcriptional and posttranscriptional regulation of gene expression and the modulation of RNA stability and translation under stress conditions [1,4–7].

## 2. Small RNAs (sRNAs)

**Figure 1:**
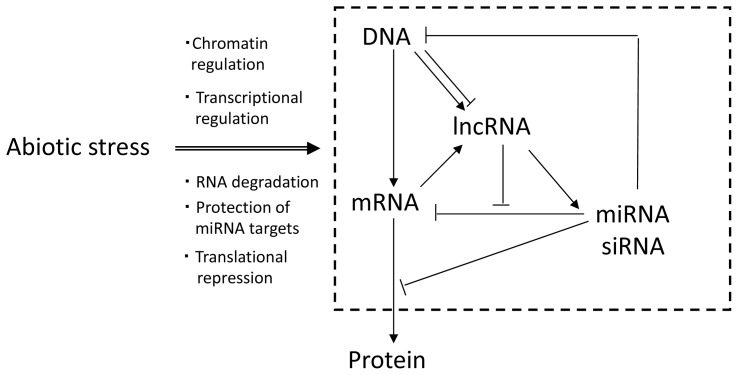
Regulation of ncRNAs in abiotic stress responses. ncRNAs (lncRNAs, miRNAs and siRNAs, *etc.*) are generated in response to abiotic stress, such as drought, low-temperature, heat and high-salinity. The ncRNAs are involved in various types of regulation, such as chromatin regulation, transcriptional regulation, RNA degradation, protection of miRNA targets, translational repression.

sRNAs are known to have major functional roles in eukaryotic gene regulation. In plants, knowledge regarding the biogenesis and mechanisms of action of sRNA classes including microRNAs (miRNAs), transcriptional gene silencing (TGS)-related heterochromatic small interfering RNAs (siRNAs), trans-acting siRNAs (ta-siRNAs) and natural antisense transcript siRNAs (nat-siRNA) has been primarily gained through *Arabidopsis* studies (Figure 1; Table 1). These sRNAs are loaded into RNA-induced silencing complexes (RISC) and negatively regulate the expression of their target genes by affecting mRNA levels, chromatin remodeling and DNA methylation.

### 2.1. Micro RNAs (miRNAs)

Many stress-responsive miRNAs have been identified in various plants [6,8]. miRNAs are generated from pri-miRNAs that are transcribed by RNA polymerase II and the mature miRNAs guide to cleavage target mRNAs [9]. Therefore, the expression levels of pri-miRNAs are regulated by *cis*-elements in a similar manner as protein-coding genes. An abundance of positive cold stress-related *cis*-regulatory elements, such as the Dehydration Responsive Element/Low Temperature Responsive Element (DRE/LTRE) -core (A/GCCGAC) [10], Abscisic Acid-Responsive Element (ABRE) -core (ACGTGG/TC) [11] and W-box (TTGAC) [12] are found in the promoter region of several cold-inducible *MIRNA* genes in *Arabidopsis* [13]. Several miRNAs that are upregulated in response to various abiotic stresses, including cold [14], dehydration [14,15], salinity [14] and nutrient deficiency [16] have been reported.

An *Arabidopsis* miRNA involved in the detoxification of reactive oxygen species (ROS) has been reported [17]. Expression of miR398 was found to be decreased by copper (Cu(II)), which is an essential nutrient in photosynthesis and response to oxidative stress and abiotic stresses [18]. miR398 was also shown to suppress superoxide dismutase (SOD) and Cu/Zinc (Zn) superoxide dismutases (CSDs) under low Cu. Interestingly, it was reported as a rare instance in plants that miR398 not only digests *CSD1* and *CSD2* mRNAs but also negatively regulated the translation of their protein products [19]. This miRNA-dependent translational repression is effected in part by the ARGONAUTE proteins AGO1 and AGO10 [19]. It also requires the activity of the microtubule-severing enzyme katanin and the de-capping component VARICOSE (VCS)/Ge-1, as recently suggested from animal studies. *katanin1* and *vcs1* mutants that did not affect *CSD2* mRNA accumulation, exhibited an overaccumulation of CSD2 proteins under low Cu (II) conditions in comparison to WT plants. However, under high Cu (II) conditions, this trend was not observed [19]. This result suggested that the translational inhibition of miR398 had an important role in the regulation of CSD expression under low Cu (II) condition.

Several miRNAs function in the maintenance of phytohormone signaling during exposure to abiotic stress. An miR168-mediated feedback regulatory loop regulates AGO1 homeostasis in ABA and abiotic stress responses in *Arabidopsis* [20]. *MIR168a*-overexpressing plants and *ago1* loss-of-function mutants showed ABA hypersensitivity and drought tolerance, while the *mir168a* mutants showed ABA hyposensitivity and drought hypersensitivity [20]. The promoter of *MIR168a* has four ABREs, suggesting that expression of *MIR168a* was directly induced by ABA. Although promoter activity of *AGO1* was induced by ABA, *AGO1* transcripts were negatively regulated by ABA-induced miR168a; and as a result, mRNA was maintained at a steady level during the stress response. These results suggest that a complex crosstalk exists between the global regulation of miRNA metabolism and ABA signaling functions to enable fine-tuning of abiotic stress response.

In another example of miRNA-phytohormone crosstalk in abiotic stress response, miR160 regulates the expression of *Auxin Response Factors* (*ARF10*, *ARF16* and *ARF17*) [21]. *Arabidopsis* plants expressing miR160-resistant *ARF10* not only showed an abnormal leaf shape but also showed hypersensitivity to ABA. Plants overexpressing miR160 showed hyposensitivity to ABA. In addition, other miRNAs targeting auxin signaling factors were also induced in response to abiotic stress [14]. Expression of miR393 was up-regulated by dehydration, salt and cold stresses and ABA [8]. A miR393 target gene, *TIR1*, an auxin receptor, is involved in the response to salt and oxidative stresses [22]. The promoter of miR167 contains ABREs, indicating their own regulation by ABA [14]. *ARF6* and *ARF8*, which are targeted by miR167, are regulators of female and male reproduction [23]. *TAS3*-siRNA also regulates auxin signaling [24]. It is thought that miRNAs targeting auxin signaling function as mediators that connect abiotic signaling with development [7,25]. These results suggest that multistep regulation by miRNAs is required for the correct adjustment of gene expression under abiotic stress.

Nutrient deficiency under abiotic stress is known to induce or suppress various miRNAs that regulate nutrient metabolism. Previous studies have demonstrated that the expression of miR395 was increased by sulfate starvation [26]. This specific miRNA suppresses ATP sulfurylases as target mRNAs, thus resulting in catalysis of the first step of inorganic sulfate assimilation [8]. A phosphate starvation-inducible miRNA (miR399) regulates Pi homeostasis by regulating the expression of *UBC24* mRNA encoding an ubiquitin-conjugating E2 enzyme [16,27]. miR399 functions as a positive regulator of Pi uptake and translocation. In addition to miR399 regulating *UBC24* expression, the cleavage activity of miR399 is suppressed by a long intergenic ncRNA, Induced by Phosphate Starvation 1 (IPS1) [28]. A detailed description is provided at a later point in this chapter.

### 2.2. Small Interfering RNAs (siRNAs)

Recent studies have reported that small interfering RNAs (siRNAs) function in stress responses. These siRNAs are generated from long double strand RNAs through various biological processes [29].

A specific type of siRNA that is involved in RNA-directed DNA methylation suppresses the activation of retrotransposons under heat stress [30]. The transcriptional gene silencing (TGS)-related heterochromatic siRNAs are generated from RNA Polymerase IV (PolIV)-derived transcripts in repetitive DNA sequences and heterochromatin. After heat stress, a *Copia*-type retrotransposon in *Arabidopsis*, named *ONSEN*, becomes transcriptionally active and has been shown to result in the synthesis of extrachromosomal DNA copies in the siRNA-mediated silencing deficient mutant *nrpd1* (*nrpd1a*), which is the largest subcomponent of PolIV. Heat-induced expression and transgenerational retrotransposition of *ONSEN* were suppressed by siRNA-mediated silencing. It was also reported that abiotic stresses changed the genome-wide DNA methylation status across multiple generations [31].

*Trans*-acting siRNAs (ta-siRNA) can be classified as a specialized case of siRNAs in plants. These siRNAs are generated from dsRNAs, which are generated from miR173, miR390 and miR828 -cleaved lncRNAs [32,33]. The long antisense non-coding RNAs (lancRNAs) of these dsRNAs were synthesized by RNA-dependent RNA polymerase 6 (RDR6), those dsRNAs were positive candidates producing siRNAs [34]. The tight feedback regulation between miR390, *TAS3* ta-siRNA and ARF4, which is a target of *TAS3* ta-siRNA, was required for lateral root initiation [24]. Although ta-siRNA expression has not been found to change in response to abiotic stress in *Arabidopsis*, the expression of a *rice RDR6 homolog*, which produces dsRNAs from cleaved *TAS* RNAs, was induced by ABA in rice [35]. These results imply that *TAS3* ta-siRNA is involved in dynamic changes of root architecture during exposure to abiotic stress [36].

Borsani *et al.* 2005 reported that natural antisense transcript small interfering RNA (nat-siRNAs) were generated from dsRNAs produced from natural *cis*-antisense gene pairs of Δ*1-Pyrroline-5-Carboxylate Dehydrogenase* (*P5CDH*) and a high-salinity-stress inducible gene of unknown function (*SRO5*) during high-salinity stress [37]. Recent genome-wide analysis reported an accumulation of sRNAs in their overlapping region, suggesting the occurrence of an RNA interference event [38]. However, the biological process of generating nat-siRNAs is not completely understood at this time [39,40].

## 3. Long Non-Coding RNAs (lncRNAs)

Genome-wide tiling arrays and high-throughput sequencing have identified a vast amount of lncRNAs in plants [4,41–45]. These lncRNAs may represent alternatively spliced forms of known genes [46], products of antisense RNAs [4,38,47,48], double stranded RNAs [49], retained introns [46,50], short open reading frame [34,51,52], RNA polymerase III-derived RNAs [53] and RNA decoys mimicking miRNA targets [28]. In this manuscript, we classified the lncRNAs into long antisense non-coding RNAs (lancRNAs) and long intergenic non-coding RNAs (lincRNAs) based on its genomic locations (Figure 1; Table 1). These RNAs have various modifications that depend on each biosynthetic process.

### 3.1. Long Antisense Non-Coding RNAs (lancRNAs)

Over the past decade, genome-wide transcriptome analyses confirmed that approximately 30% of all annotated genes exhibited significant lancRNA expression in *Arabidopsis* [4,41]. These data regarding lancRNA expression are consistent with results from other organisms such as fly, human, and rice [54]. Expression profiles of lancRNAs in response to environmental stresses have been extensively characterized by an *Arabidopsis* tiling array analysis [4]. A certain type of lancRNAs belongs to a pair of fully overlapping sense–antisense transcripts (fSATs) in which the lancRNAs exist within the protein-coding gene regions in opposing orientations. The expression of these sense and antisense RNA transcripts are stress-responsive. On the other hand, partially overlapping sense–antisense transcripts (pSATs) do not exhibit synchronous expression patterns. These observations suggest that lancRNA were generated through multiple biosynthetic processes.

A type of lancRNA is co-expressed with sense protein-coding RNAs [4]. A large *Arabidopsis* tiling array analysis confirmed that more than 6000 lancRNAs were classified into the fSATs category. Interestingly, a significant linear correlation between the expression ratios (abiotic stress treated/untreated) of the sense transcripts and the ratios of the lancRNAs was observed in the fSATs. The *RD29A* and *CYP707A1* lancRNAs that were simultaneously accumulated with sense mRNAs, were accumulated by drought- and ABA treatments. Some of the lancRNAs that were identified contained complementary sequences to those of the sense mRNAs [4], indicating that lancRNA expression is dependent upon sense mRNAs.

Co-expression of sense RNA and lancRNA of a transgene was reported as a trigger for sense post-trancriptional gene silencing (S-PTGS) [55]. In the S-PTGS process, RNA-dependent RNA polymerase 6 (RDR6), one of six *Arabidopsis* RDRs, generates antisense RNAs from non-canonical sense RNAs of transgenes with aberrant features, such as non-cap structure of 5′ end or poly(A) tail, to generate double stranded RNA (dsRNA) [56]. dsRNA-seq analysis showed that dsRNAs of more than 100 loci were reduced in *rdr6* in relative comparison to WT [57]. RNA-seq analysis of sRNAs also revealed that mutations of *ABH1* and *EIN5* (*XRN4*), which are involved in mRNA processing and mRNA degradation, respectively, affect the level of sRNAs mapped on the antisense strand of endogenous protein-coding genes [58]. The *xrn4* mutant was also screened as an enhancer of transgene silencing [59]. These results supported the observation that a certain type of lancRNA was generated from non-canonical sense RNA. Recent studies revealed that *Werner Exonuclease* (*WEX*), *Silencing Defective3* (*SDE3*), *DCL2*, *DCL4*, *Nuclear RNA Polymerase IVa* (*NRPD1a*), *RDR2* and *CLASSY1* were involved in S-PTGS and downstream PTGS [60–65]. Since S-PTGS-related siRNAs are generated from dsRNAs, these results also indicate that a certain type of lancRNA is synthesized from mRNA templates via a complex amplification pathway.

It is possible that the expression of lancRNA could serve as a functional link to the chromatin regulation of epigenetic silencing [48]. *Cold Induced Long Antisense Intragenic RNA* (*COOLAIR*) in the *FLC* locus is a well-characterized example of this in *Arabidopsis* [47,66]. Exposure of plants to low temperature treatment for a 2-week period resulted in a high level of *COOLAIR* expression. Several weeks after the induction of *COOLAIR*, the transcription of *FLC* was significantly decreased. During this period, tri-methylated histone H3 Lys27 (H3K27me3) levels progressively increased at a region around the transcription start site [67]. The level of H3K27me3 spreading the gene body was required to maintain the repression of *FLC* transcription after plants were returned to warm conditions [68]. *COOLAIR* has been suggested to be required for a plant homeodomain-polycomb repressive complex 2 (PHD-PRC2) located at a tightly localized nucleation region within *FLC*. Consequently, this results in an increase in H3K27me3 levels at the *FLC* locus [64]. Although further analysis is necessary to elucidate the role of *COOLAIR* and its epigenetic silencing of *FLC* during the short period of vernalization [69,70], a reporter gene that was fused with *COOLAIR* was shown and confirmed to be capable of causing cold-induced silencing [47].

The second class of lncRNA, *COLDAIR*, is transcribed from a region within the first intron of *FLC* on the sense strand [71]. The *COLDAIR* transcript has been shown to interact with PRC2 and its abundance also increased during vernalization. Reduction of *COLDAIR* transcript levels by RNAi confirmed that it is not required for the initial repression of *FLC* but is necessary for subsequent maintenance of repression. These results showed that the interaction of lncRNAs and the chromatin modification complex mediates cold-inducible epigenetic regulation. Based upon bioinformatic analyses comparing lancRNAs and chromatin status, it was recently hypothesized that a type of lancRNAs from the genome was repressed by cytosine methylation and H3K36me [48]. This prediction proposed that an interaction between chromatin regulation and a certain type of lancRNAs functions in genome regulation.

A recent strand-specific RNA-seq study showed that approximately 1300 ncRNA loci exist in the antisense strand of protein-coding genes in *Arabidopsis* [48]. The global ratio between sense and antisense tags in exons in this study was 0.01, which was similar to a previous strand-specific RNA-seq analysis using floral tissue [43,48]. A comparison between RNA-seq and genome-wide tiling array data showed that one-half to two-thirds of the sense–antisense transcripts were only represented in one experiment [48]. Since the expression levels of lancRNAs are low compared to sense transcripts, it is possible that this difference between the two techniques may reflect technical limitations inherent to these methods. Future transcriptome analyses are required to increase our understanding of non-canonical transcripts and to clarify the types of lancRNAs that function in molecular signals, RNA decoys, guides, and scaffolds [72].

### 3.2. Long Intergenic Non-Coding RNAs (lincRNAs)

Several transcriptome analyses have reported that more than 1000 lincRNA loci exist between protein-coding genes in *Arabidopsis* [4,41–45]. A part of these lincRNAs was transcribed from the methylated DNA regions by RNA polymerase IV and are thought to be positive candidates that generate into TGS-related heterochromatic siRNAs that guide DNA methylation [64].

Another type of lincRNA is transcribed by RNA Polymerase III. *In silico* genome sequence analysis predicted 20 novel ncRNA candidates [53]. A specific Pol III-derived ncRNA (*AtR18*) responded negatively to hypoxic stress and this regulation was evidently different from that of *U6* snRNA. Specifically, *AtR18* was not processed into a smaller fragment and no small open reading frames (sORFs) were included. Short interspersed elements (SINEs) and 7SL (signal recognition particle) RNA for protein trafficking are known as Pol III-derived RNA, with exception of the canonical functional RNA [73,74].

Many sORFs that have not been annotated as protein-coding genes have been identified as expressed genes during developmental or environmental conditions [51,52]. A specific sORF (*npc536*) has a large dynamic variation of expression across a wide range of tissue and hormonal, biotic, or abiotic treatment [34]. *npc536* exists in the antisense strand of a Golgi-transport complex related protein. *35S::npc536* transformants displayed heightened root growth under salt stress conditions. However, the biological function of this RNA is not still clearly understood at this time.

LincRNA is capable of regulating the cleavage activity of miRNA as a target mRNA decoy. *IPS1* is a lincRNA, which was found to be induced by phosphate starvation [28,75]. Although *Arabidopsis IPS1*, *AT4-1*, *AT4-2* and family members in other species share little sequence conservation with the predicted ORFs, high conservation was observed with a 22-nt sequence located in the 3′ half of the transcript [75]. This observation suggests that the *IPS1* family contains the sequence that is targeted by miRNA and it does not function as a short peptide. The 22-nt sequence of *IPS1* is not perfectly complementary to miR399 due to a mismatch occurring at the 10–11th position [28]. Although miR399 was capable of hybridizing to *IPS1* transcript, there has been no indication of cleavage via miR399. On a whole plant level, phosphate starvation resulted in the induction of *IPS1*. As a result of the phosphate starvation, the miR399 target (*UBC24* mRNA) accumulated. miR399 has been characterized as a phosphate starvation inducible-miRNA and a positive regulator of Pi-uptake. Interestingly, two studies implied that miR399 may function as a long-distance signal from shoots to suppress *UBC24* expression in roots [76,77]. Taken together, it is reasonable to consider that the complex interactions between *IPS1*, miR399 and *UBC24* function to maintain spatial homeostasis of Pi. Computational analyses predicted the occurrence of these possible target mimics in rice and *Arabidopsis*, suggesting that RNA decoys (miRNA target mimic) are conserved and functional contributors to the regulation of RNA [78].

## 4. Conclusions and Perspectives

Whole genome transcriptome analyses of high-density microarrays and high throughput sequencing have expanded our understanding of a novel research area pertaining to networks, which govern abiotic stress responses. Despite the extensive body of published information regarding various types of ncRNAs, their association to the adaptation and response of plants to abiotic stress is not completely understood at this time. Recent advances in whole transcriptome analyses have enabled us to gain a greater understanding regarding the mechanisms of transcriptional and posttranscriptional regulation through ncRNAs, as well as sRNAs and lancRNAs. Functional analyses of miRNAs have deciphered and unraveled complex feedback loop regulations between miRNA and target genes. In addition, the characterized interaction between miR399, *IPS1* and *UBC24* has been shown to be a novel type of RNA interaction system in which a lincRNA reduced a cleavage activity of phosphate starvation-inducible miRNA and regulated a target mRNA. In addition, ncRNAs are also linked to chromatin regulation. The studies regarding epigenetics in the *FLC* locus enabled a hypothesis to be made which suggests that an interaction of lancRNA and chromatin modification complex mediates cold-inducible epigenetic regulation. Overall, these data have revealed a complex interaction between transcriptional regulators which function to fine-tune responses to various environmental stimuli. For the future, it will be essential to investigate the biological regulations of ncRNAs in order to enable scientists to completely elucidate the entire picture of gene regulation networks in stress response.

## Figures and Tables

**Table 1:** Classification of non-coding RNAs (ncRNAs) (1) involved in abiotic stress responses.

**Small ncRNAs (sRNAs)**
micro RNAs (miRNAs) small interfering RNAs (siRNAs) TGS-related heterochromatic siRNAs *trans*-acting siRNAs (ta-siRNAs) natural antisense transcript siRNAs (nat-siRNA) others
**Long ncRNAs (lncRNAs)**
long antisense non-coding RNAs (lancRNAs) lancRNAs generated during biogenesis of ta-siRNAs and S-PTGS-related siRNAs lancRNAs involved in epigenetic silencing lancRNAs co-expressed with sense-stranded protein-coding mRNAs others long intergenic non-coding RNAs (lincRNAs) RNAs generated from small open reading frame regions RNA decoys mimicking miRNA targets RNAs transcribed by Pol III RNAs transcribed by Pol IV during biogenesis of TGS-related heterochromaticsiRNAs others

(1) ncRNAs are classified except for well-known housekeeping ncRNAs, such as rRNAs, tRNAs, snRNAs and snoRNAs.
